# Cell Death Mechanisms at the Maternal-Fetal Interface: Insights into the Role of Granulysin

**DOI:** 10.1155/2012/180272

**Published:** 2011-09-08

**Authors:** Danijela Veljkovic Vujaklija, Sonja Sucic, Tamara Gulic, Marin Dominovic, Daniel Rukavina

**Affiliations:** ^1^Department of Physiology and Immunology, Medical Faculty, University of Rijeka, 51000 Rijeka, Croatia; ^2^Department of Radiology, Clinical Hospital Center Rijeka, 51000 Rijeka, Croatia; ^3^Center for Physiology and Pharmacology, Institute of Pharmacology, Medical University Vienna, 1090 Vienna, Austria

## Abstract

During mammal pregnancy, a sensitive balance between hormones, cytokines, humoral factors, and local cellular interactions must be established. Cytotoxic cells infiltrating the decidua are heavily equipped with cytolytic molecules, in particular perforin and granulysin. Granulysin is especially abundant in NK cells which are able to spontaneously secrete high quantities of granulysin. Besides being a potent bactericidal and tumoricidal molecule, granulysin is also found to be a chemoattractant and a proinflammatory molecule. The precise role(s) of granulysin at the maternal-fetal interface has not been elucidated yet. It is possible that it behaves as a double-edged sword simultaneously acting as an immunomodulatory and a host defense molecule protecting both the mother and the fetus from a wide spectrum of pathogens, and on the other hand, in case of an NK cell activation, acting as an effector molecule causing the apoptosis of semiallograft trophoblast cells and consequently leading to various pregnancy disorders or pregnancy loss.

## 1. Introduction

Pregnancy is a unique event whereby the fetal semiallogenic trophoblast cells develop a close contact with the mother's fully competent immune system. In order for implantation to be successful, the uterus must undergo specific tissue transformation to establish a sensitive cytokine and hormonal balance [1]. Decidualization of the human endometrium following embryo implantation is normally associated with massive recruitment of distinct CD56^bright^CD16^−^ natural killer (NK) cells [2, 3]. Sharing common cytotoxic pathways with cytotoxic T lymphocytes (CTL), these cells collectively play a vital role in the maintenance of pregnancy and protection against numerous pathogens. There are two main pathways of lymphocyte-mediated cytotoxicity: (i) granule exocytosis pathway and (ii) death receptor signaling pathways [4, 5].

## 2. Granule Exocytosis Pathway

**Figure 1:**
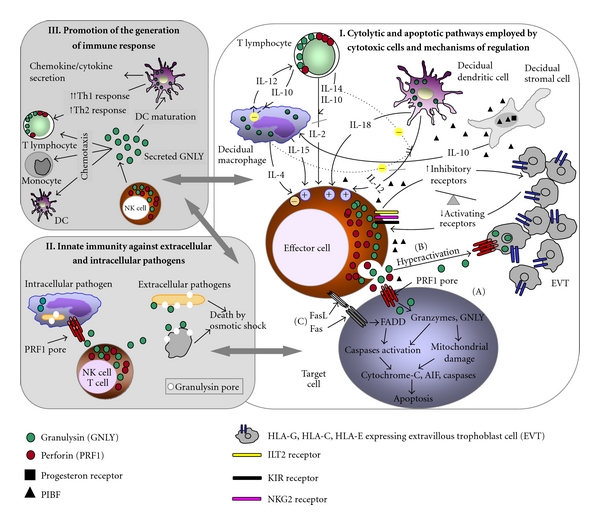
Cytolytic and apoptotic pathways at the maternal-fetal interface employed by cytotoxic cells and mechanisms of regulation with the particular insight to the role(s) played by granulysin.

The granule exocytosis pathway is mediated *via* release of cytotoxic granules directly into the synapse between the effector and their target cells. The granules released contain a pore-forming protein, perforin, an apoptotic/cytolytic protein, granulysin, and a family of serine proteases, granzymes [5–7]. Upon release, perforin inserts itself into the plasma membrane of the target cells, polymerises, and subsequently forms cylindrical pores to allow granzymes, in particular granzyme B, and granulysin to enter the cell and initiate apoptosis [7]. Perforin is essential for the release of granzymes from the cytolytic granules although, as for granulysin, perforin is not necessary for their entry into the target cell [8, 9] (Figure 1-I(A)). 

The cytolytic machinery of decidual lymphocytes is optimally primed to kill, but at the same time, it is precisely regulated by humoral factors, cytokines (predominantly Th2 over Th1 cytokines), and local cellular interactions, to prevent accidental activation that could lead to the termination of pregnancy [10, 11]. More than 90% of all decidual CD56^bright^CD16^−^ cells express perforin, which is present here in higher levels than in any other tissue under physiological or pathological conditions [12]. Nonstimulated decidual NK cells are not cytotoxic, but following an *in vitro *stimulation with Th1 cytokine interleukin (IL)-2 which is in early pregnancy decidua virtually absent, they acquire lymphokine-activated killing (LAK) activity against trophoblast cells [13, 14]. Earlier results from our laboratory support these findings, showing that IL-2 increases perforin gene and protein expression in decidual lymphocytes [4]. Cytolytic activity of decidual lymphocytes is also upregulated by IL-15, IL-18, and IL-12, all acting by increasing perforin and FasL expression [4, 15–17] (Figure 1-I(A), (B)). 

Conversely, perforin expression in decidual lymphocytes is downregulated by progesteron, either directly or *via *progesterone-induced blocking factor (PIBF) [10, 18]. The lack of cytolytic activity of decidual NK cells against trophoblasts could also be attributed to inhibitory interactions of nonclassical class I HLA molecules (HLA-G and HLA-E) and low polymorphic classical class I HLA molecule HLA-C expressed by extravillous trophoblast cells with their respective inhibitory receptors immunoglobulin-like transcript 2 (ILT2), C-type lectin family receptor CD94/NKG2A, and killer Ig-like receptors (KIRs) expressed on NK cell surface [19–21]. In normal early pregnancy, specific interaction between NKG2A and HLA-E mediates a dominant negative signal *in vivo*, to prevent perforin recruitment and potentially harmful cytotoxicity [22]. However, decidual NK cells also express a series of activating receptors, such as NKG2D, but also natural killer cytotoxicity receptors NKp30, NKp44, and NKp46, whose overengagement could trigger decidual NK cell cytotoxic activity [22–24].

## 3. Death Receptor Pathway

The death receptor pathway is mediated by Fas/FasL, tumor necrosis factor, (TNF-) receptor family, and TNF-related apoptosis-inducing ligand (TRAIL). Fas receptor is ubiquitously expressed within most healthy cells, including NK cells, activated T and B lymphocytes, and dendritic cells [25, 26]. FasL is in human pregnant uterus stored in the intracellular granules of T and NK cells, localized to the sites of close contact with the placental tissue, and can be exocytosed upon nonspecific activation of these cells [27, 28] (Figure 1-I(C)). The ligation of Fas and FasL results in a classical caspase-dependent apoptosis involving the adaptor molecule Fas-associated death domain (FADD) protein [6] (Figure 1-I(C)). In the villous part of the placenta where the Fas/FasL interaction seems to be involved in the regulation of placental growth, FasL is mostly located on cytotrophoblast cells, whereas syncytiotrophoblast cells express low FasL levels. In contrast, interstitial trophoblast cells, which are in close contact with the maternal leukocytes, strongly express FasL, but not Fas. The abundant expression of FasL on extravillous trophoblast probably serves as a defense mechanism against activated maternal leukocytes [4, 29]. Th1 cytokines, IFN-*γ* and TNF-*α*, promote Fas expression on trophoblast, thus making them susceptible to Fas/FasL-mediated apoptosis by activated maternal lymphocytes, whereas Th2 cytokines increase the resistance of trophoblast cells to Fas-mediated apoptosis [30]. TRAIL is a TNF-protein family member, structurally and functionally similar to FasL. It is expressed by syncytiotrophoblast and, along with FasL, is likely involved in the maintenance of immunoprivileged conditions at the maternal-fetal interface [31, 32]. The exact role of perforin and death receptor interactions during human pregnancy has not been elucidated yet, but it is believed that they serve as a molecular weapon of uterine NK cells by (i) playing an important role in the acceptance of the fetus and the control of trophoblast invasion and (ii) being crucial for defense against microbe-infected, stressed, or malignant cells.

## 4. Granulysin-Mediated Cell Death

Another protein that could be vital for the maintenance of normal pregnancy is granulysin. Granulysin is a recently discovered protein, found to be highly expressed in human NK cells and activated CTLs. Over the last decade, granulysin has been a protein of significant scientific interest, mainly due to the cytolytic activity it exhibits on the numerous microbes ranging from extracellular and intracellular bacteria to fungi and parasites, but also due to its tumoricidal activity [9, 33]. 

The granulysin protein can be found in two forms: 9 and 15 kDa [34]. The cytolytic 9 kDa form is active, and it is achieved *via* a posttranslational proteolytic cleavage at the amino and carboxyl terminals of the 15 kDa precursor granulysin protein [35]. However, Chung and colleagues [36] have recently shown* in vitro* cytotoxic effects even for the 15 kDa granulysin protein. Granulysin protein is expressed in various cell subsets, such as the activated CD4^+^ and CD8^+^ T lymphocytes, NK cells and in activated, but not in resting, CTLs [37], NKT cells [38] as well as decidual *γδ* T cells [39], and tuberculosis-specific V*γ*9/V*δ*2 T cells [40]. 

Granulysin belongs to the family of saposin-like lipid binding proteins, with the highest level of homology to NK-lysin. The three-dimensional structure obtained from X-ray crystallography of recombinant granulysin reveals that it is composed of five *α*-helices, separated by short-loop regions [41, 42]. It is believed that the lytic activity of granulysin can be explained by its cationic ampholytic structure based on which granulysin disrupts bacterial membrane which generally contains negatively charged lipids and hence mediates bactericidal activity by osmotic shock [43, 44] (Figure 1-II), In contrast, granulysin does not permeabilize target cell membranes when bound to lipid rafts or phospholipid membranes with eukaryotic lipid composition, but it can be internalized into such cells (e.g., infected eukaryotic cells) by lipid rafts and delivered to the early sorting endosomes which afterwards fuse with bacteria-containing phagosomes, where finally the lysis of bacteria is induced [45–47]. The data on the role of perforin in granulysin uptake in infected eukaryotic cells is inconclusive. Stenger et al. [9] reported that in order to kill intracellular pathogens, granulysin requires perforin as a cofactor to enter the host cells, whereas Walch et al. [47] report that perforin promotes granulysin-mediated bacteriolysis not by the formation of stable pores that allow passive diffusion of granulysin but rather by an increase in endosome-phagosomes fusion triggered by an intracellular Ca^2+^ rise (Figure 1-II).

Death of the tumor cells is caused by granulysin-initiated apoptosis. Upon binding to the membrane based on charges, granulysin activates Sphingomyelinase followed by a slow increase in ceramide concentration or induces an increase in intracellular calcium and efflux of intracellular potassium [48–50]. Both pathways are linked with fast mitochondrial membrane damage, which is the key step in granulysin-induced apoptosis [51, 52]. This results with release of cytochrome C and apoptosis-inducing factor A [53] followed by activation of caspases and endonucleases and finally the cell death by apoptosis [49, 54] (Figure 1-I(A)). The latest report by Saini et al. [50] shows that NK cell-delivered granulysin and recombinant granulysin induce target cell death through distinct pathways. Granulysin delivered by NK cells does not cause mitochondrial damage or activates either caspase-3 or caspase-9 in target cells, whereas recombinant 9-kDa granulysin activates these pathways. Unlike recombinant granulysin, NK cell-delivered granulysin causes both endoplasmic reticulum stress and caspase-7 activation in target cells [50]. 

Recently, granulysin has been characterized as the first lymphocyte-derived protein found to act as an alarmin, capable of promoting antigen-presenting cell (APC) recruitment and antigen-specific immune response [55]. At nanomolar concentrations, predominantly 15 kDa granulysin acts as a chemoattractant for immune cells, such as monocytes, NK cells, and CD4^+^ and CD8^+^ memory T-lymphocytes [55, 56] and has a proinflammatory effect due to its ability to activate monocytes to produce cytokines, such as IFN-*γ* and TNF-*α*, and chemokines, such as MCP and RANTES [56] (Figure 1-III). The latest results by Castiello et al. [57] revealed that *in vitro,* 15 kDa granulysin induces immune response, chemotaxis, and cell adhesion genes in human peripheral blood monocytes. Further, granulysin-treated monocytes upregulated genes involved in the activation of pathways related to fundamental dendritic cell functions, such as costimulation of T-cell activation and Th1 development, for example, upregulation of genes in the IL-12 and STAT4-dependent signaling [57]. 

Therefore, granulysin presents a novel cytolytic molecule with immense biomedical and therapeutic potential [58]. Besides its ability to kill bacteria, fungi, and parasites, granulysin can block viral replication and trigger apoptosis in infected cells [59]. The fact that it can kill many deadly pathogens makes granulysin, and its recombinant peptide derivatives, an attractive target in the development of novel classes of antibiotics, with less resistance observed for most currently available therapies. In addition, 15 kDa granulysin holds promise for therapeutic applications aimed at the activation of the immune response.

## 5. Granulysin at the Maternal-Foetal Interface: A Double-Edged Sword

Granulysin mRNA is expressed in human endometrium of nonpregnant women with highest expression in the late secretory phase of the menstrual cycle, correlating with the increase in uterine NK cell number towards the end of the secretory phase [60]. Granulysin expression is most likely under the control of progesteron since the antiprogestin treatment of secretory endometrial explants decreased the granulysin mRNA expression [60]. Perforin exhibits a similar pattern of expression during menstrual cycle with massive recruitment of perforin-positive cells following the progestin-induced endometrial decidualization [61]. The expression of granulysin mRNA in early pregnancy and second trimester placentas increases further, whilst its expression is downregulated at term (37–40 weeks) placentas [62]. 

The granulysin (GNLY) gene expression in secretory phase human endometrium was localized to uterine NK cells scattered around the stroma and surrounding the glandular epithelium [63]. Our recent results show that first trimester pregnancy decidual tissue is diffusely infiltrated by granulysin-positive cells which accumulate around uterine glands and blood vessels [64, 65]. Granulysin protein was also found in peripheral blood lymphocytes of pregnant women but in significantly lower amounts [64]. Immunophenotipisation of first trimester pregnancy decidual lymphocytes revealed that over 85% of CD56^+^CD3^−^ NK cells and 75% of CD56^+^CD3^+^ NKT cells express granulysin. Similar to decidual, peripheral blood NK and NKT cells also highly express granulysin. At gene level, granulysin is highly expressed in all decidual lymphocyte subpopulations, especially in CD56^+^ cells. In comparison to other cytolytic mediators (perforin, FasL, TRAIL), granulysin mRNA is present in significantly higher levels in CD56^+^ and CD56^+^CD3^+^ cells, but in T lymphocytes only, no differences between granulysin and perforin mRNA levels were observed (unpublished data, manuscript in preparation). It is known that NK cells constitutively express high levels of granulysin, whereas granulysin expression in T lymphocytes is inducible after mitogen or alloantigen stimulation [66, 67]. Surprisingly, more than half of decidual T lymphocytes (approximately 58%) express granulysin protein, whereas only few percent of peripheral blood T lymphocytes express granulysin [64]. The striking increase of the percentage of granulysin-positive decidual T lymphocytes suggests that these cells are locally activated, even in the early phase of pregnancy, probably after the contact with semiallogenic fetal cells and mature dendritic cells. Hence, these cells could play a protective role against different pathogens at the maternal-fetal interface by delivering granulysin which in turn either reduces the viability of the extracellular or intracellular pathogens as described previously, possibly leaving the host cell intact [9, 45]. Moreover, Stenger et al. [9] correlated the CTLs ability to reduce the viability of intracellular pathogens with granulysin expression in these cells. The protective role of granulysin was further supported by findings by Fleming and coworkers [60] which stated that granulysin enhances the innate immune capacity of endometrium since women taking the oral combined hormonal contraceptive pill or wearing a levonorgestrel intrauterine system have a significantly lower expression of granulysin mRNA in the late secretory phase of menstrual cycle and are more susceptible to various infections. Granulysin was also found to be expressed by *γδ* T cells of human early-pregnancy decidua, further supporting the protective role of granulysin as an innate immunity molecule in early pregnancy [39]. 

Granulysin could also have a very important immunomodulatory role at the maternal-fetal interface (Figure 1-III). Recently, Tewary et al. [55] reported that both 9 kDa and 15 kDa forms of granulysin induce the migration as well as functional and phenotypical maturation of human monocyte-derived dendritic cells (Mo-DC) by upregulation of CD80, CD83, and MHC class II molecules. Also, it induces higher production of IL-6, IL-8, IL-12, IL-10, and TNF-*α* by Mo-DC and enhances their capacity to stimulate allogeneic T-cell proliferation* in vitro* [55]. The 15 kDa granulysin might also play an important role in activating the immune system in response to pathogens by inducing monocytes to recruit other immune cells by upregulating a wide group of genes responsible for chemotaxis of different immune cells, such as neutrophils (CXCL1, CXCL3), T cells (CXCL11, CXCL12, CCL20, and CCR7), monocytes (CCL2, CCL20), macrophages, and dendritic cells (NRP2, SEMA3A) [57]. Cognition that granulysin acts as a chemoattractant to such various cells types opens the question whether granulysin produced by decidual NK cells could also mobilize invasive trophoblast cells.

Our latest results revealed that uterine CD56^+^ cells spontaneously secrete high levels of granulysin [64]. The amounts of granulysin secreted by decidual CD56^+^ cells were almost equivalent to values at which the peak migratory response of dendritic cells was observed in the study by Tewary et al. [55]. In contrast, peripheral blood CD56^+^ cells virtually do not secrete granulysin after 2 hrs in culture. After 18 hrs in culture, they start secreting very low levels of granulysin, which are well in agreement with studies by Ogawa et al. [68] and Sakai et al. [69]. In our opinion, considering that the female reproductive tract is potentially exposed to wide range of pathogens, and infections of genital tract during pregnancy may have severe consequences such as miscarriage or preterm birth, the spontaneous secretion of high levels of granulysin probably serves as a mechanism of protection of both mother and fetoplacental unit [64, 70, 71]. After being spontaneously secreted into local environment, granulysin might be able to promptly kill various pathogens without causing significant damage to normal cells, whereas simultaneously through its effects on APCs and other immune cells it might participate in the regulation of the adaptive immune response (Figures 1-II and 1-III). Moreover, decidual APCs, both dendritic cells, and macrophages, and IL-15 which is known to be produced by APC, contribute to the maintenance of high granulysin expression in decidual NK cells, thus providing continuous local protection [64]. Another important role of granulysin could be in one of key events of early pregnancy, the angiogenesis, since Langer and coworkers demonstrated that NKG5 protein (granulysin) secreted by decidual NK cells and activated T lymphocytes stimulates mitogenicity of endothelial cells and may be involved in angiogenesis [72]. 

On the other hand, several studies have shown that granulysin may be involved in different pregnancy disorders [69, 73]. Granulysin was previously associated with the development of preeclampsia as a Th1 marker. Its serum levels in preeclamptic patients were well associated with the percentage of peripheral blood Th1 cells, Th1/Th2 ratios, and the clinical outcome [69]. In cases of ectopic pregnancy, granulysin-expressing cells were virtually absent in the tubal mucosa which could explain invasiveness of trophoblast cells. Interestingly, the uterine decidua of ectopic pregnancy was simultaneously heavily infiltrated with granulysin-positive cells [65].

As mentioned, during normal pregnancy, uterine NK cells are not directed to kill trophoblast cells, but in case of excessive Th1 response due to infection or inflammation, these cells become hyperactivated and potentially cytotoxic (Figure 1-I(B)) [30, 74]. Nakashima et al. [73] have shown that granulysin contributes to apoptosis of extravillous trophoblast cells in spontaneous abortions. Granulysin secreted by CD56^bright^  uterine NK cells was detected in the cytoplasm and nuclei of apoptotic extravillous cytotrophoblast cells suggesting that granulysin may be a key substance in induction of spontaneous abortion. *In vitro* studies showed that only hyperactivated, that is, IL-2 stimulated uterine NK cells secrete granulysin which in turn actively accumulates in nuclei of extravillous trophoblast cell line. Granulysin entrance into trophoblast cells, as well as NK cell-mediated killing of target tumor cells, was shown to be dependent on perforin and cell-to-cell contact. [50, 73] (Figure 1-I(B)). Several studies have shown that HLA-G molecule inhibits natural killer cell-mediated cytotoxicity [75, 76]. In fact, our latest results show that one of the mechanisms which might prevent an excessive NK cell activation during normal pregnancy could be HLA-G mediated. Namely, decidual NK cells after the contact with HLA-G, transfected NK sensitive K562 cells express and secrete significantly lower quantities of granulysin in comparison to NK cells exposed to HLA-C-transfected K562 cells (unpublished data, manuscript in preparation). 

In light of all that is currently known, and considering that granulysin is abundantly expressed in early pregnancy decidua, especially in NK cells, we hypothesize that granulysin is a novel lethal weapon of uterine NK cells acting as a double-edged sword at the maternal-fetal interface (Figure 1). At the same time, it acts as a host defense molecule protecting both the mother and the fetus from a wide spectrum of pathogens, and as an immunomodulatory molecule by inducing chemotaxis of different immune cells, phenotypical and functional maturation of dendritic cells, and by upregulating the adaptive immune response. On the other hand, in case of NK cell activation, along with perforin, granulysin can act as an effector molecule causing the apoptosis of semiallograft trophoblast cells, consequently leading to various pregnancy disorders including spontaneous pregnancy termination, intrauterine fetal growth retardation, and preeclampsia. However, many details regarding the exact mechanisms underlying the remarkable modes of granulysin action and its physiological roles remain yet to be elucidated.

Decidual cytotoxic cells are heavily equipped with cytolytic molecules, in particular perforin and granulysin. Specific interactions between nonclassical class I MHC molecules expressed on extravillous trophoblast (EVT) cells and their inhibitory and activating receptors expressed on NK cell surface, along with antigen-presenting cells and complex network of cytokines and hormones, contribute to the maintenance of pregnancy. Th2 cytokines (IL-4, IL-10), which are predominant in early pregnancy decidua, downregulate cytolytic activity, whereas Th1 cytokines (IL-2, IL-15, IL-12, and IL-18) upregulate the same. In case of activation, the interaction between target and effector cells results in effector cell activation and directed granule exocytosis (IA). In case of hyperactivation of NK cells, that is, stimulation of IL-2, granulysin is released from cytoplasmic granules of NK cells and accumulates in the cytoplasm and nuclei of EVT cells in spontaneous abortions (IB). The ligation of FasL, expressed on NK or T-cell surface, with the death receptor Fas, on the target cell, results in caspase cascade activation (IC). All pathways result in endonuclease activation and apoptosis. At the same time, high granulysin levels might have a protective role at the maternal-fetal interface by (i) contributing to the local innate immune capacity against intracellular and extracellular pathogens (II), or (ii) acting as an immunomodulatory molecule by inducing phenotypical and functional maturation of dendritic cells, and (iii) as a proinflammatory molecule by inducing chemotaxis of different immune cells and upregulating the adaptive immune response (III).

## References

[1] Red-Horse K, Zhou Y, Genbacev O (2004). Trophoblast differentiation during embryo implantation and formation of the maternal-fetal interface. *Journal of Clinical Investigation*.

[2] Le Bouteiller P, Piccinni M-P (2008). Human NK cells in pregnant uterus: why there?. *The American Journal of Reproductive Immunology*.

[3] Trundley A, Moffett A (2004). Human uterine leukocytes and pregnancy. *Tissue Antigens*.

[4] Crncic TB, Laskarin G, Juretic K (2005). Perforin and Fas/FasL cytolytic pathways at the maternal-fetal interface. *The American Journal of Reproductive Immunology*.

[5] Chávez-Galán L, Arenas-Del Angel MC, Zenteno E, Chávez R, Lascurain R (2009). Cell death mechanisms induced by cytotoxic lymphocytes. *Cellular and Molecular Immunology*.

[6] Trapani JA, Smyth MJ (2002). Functional significance of the perforin/granzyme cell death pathway. *Nature Reviews Immunology*.

[7] Pipkin ME, Lieberman J (2007). Delivering the kiss of death: progress on understanding how perforin works. *Current Opinion in Immunology*.

[8] Motyka B, Korbutt G, Pinkoski MJ (2000). Mannose 6-phosphate/insulin-like growth factor II receptor is a death receptor for granzyme B during cytotoxic T cell-induced apoptosis. *Cell*.

[9] Stenger S, Hanson DA, Teitelbaum R (1998). An antimicrobial activity of cytolytic T cells mediated by granulysin. *Science*.

[10] Rukavina D, Podack ER (2000). Abundant perforin expression at the maternal-fetal interface: guarding the semiallogeneic transplant?. *Immunology Today*.

[11] Rukavina D, Vince G (2000). Roles of cytokines and immune cells at the interface—a workshop report. *Placenta*.

[12] Rukavina D, Rubesa G, Gudelj L, Haller H, Podack ER (1995). Characteristics of perforin expressing lymphocytes within the first trimester decidua of human pregnancy. *The American Journal of Reproductive Immunology*.

[13] King A, Birkby C, Loke YW (1989). Early human decidual cells exhibit NK activity against the K562 cell line but not against first trimester trophoblast. *Cellular Immunology*.

[14] King A, Loke YW (1990). Human trophoblast and JEG choriocarcinoma cells are sensitive to lysis by IL-2 stimulated decidual NK cells. *Cellular Immunology*.

[15] Strbo N, Laskarin G, Crncic TB (2006). Short-term cytolytic mediators’ expression in decidual lymphocytes is enhanced by interleukin-15. *The American Journal of Reproductive Immunology*.

[16] Laskarin G, Strbo N, Crncic TB (2005). Physiological role of IL-15 and IL-18 at the maternal-fetal interface. *Chemical Immunology and Allergy*.

[17] Hyodo Y, Matsui K, Hayashi N (1999). IL-18 up-regulates perforin-mediated NK activity without increasing perforin messenger RNA expression by binding to constitutively expressed IL-18 receptor. *Journal of Immunology*.

[18] Laškarin G, Štrbo N, Sotošek V (1999). Progesterone directly and indirectly affects perforin expression in cytolytic cells. *The American Journal of Reproductive Immunology*.

[19] Moretta L, Bottino C, Pende D, Vitale M, Mingari MC, Moretta A (2005). Human natural killer cells: molecular mechanisms controlling NK cell activation and tumor cell lysis. *Immunology Letters*.

[20] Hanna J, Mandelboim O (2007). When killers become builders. *Trends in Immunology*.

[21] Apps R, Murphy SP, Fernando R, Gardner L, Ahad T, Moffett A (2009). Human leucocyte antigen (HLA) expression of primary trophoblast cells and placental cell lines, determined using single antigen beads to characterize allotype specificities of anti-HLA antibodies. *Immunology*.

[22] El Costa H, Tabiasco J, Berrebi A (2009). Effector functions of human decidual NK cells in healthy early pregnancy are dependent on the specific engagement of natural cytotoxicity receptors. *Journal of Reproductive Immunology*.

[23] El Costa H, Casemayou A, Aguerre-Girr M (2008). Critical and differential roles of NKp46- and NKp30-activating receptors expressed by uterine NK cells in early pregnancy. *Journal of Immunology*.

[24] Tabiasco J, Rabot M, Aguerre-Girr M (2006). Human decidual NK cells: unique phenotype and functional properties—a review. *Placenta*.

[25] Stranges PB, Watson J, Cooper CJ (2007). Elimination of antigen-presenting cells and autoreactive T cells by Fas contributes to prevention of autoimmunity. *Immunity*.

[26] Nagata S, Golstein P (1995). The Fas death factor. *Science*.

[27] Blott EJ, Bossi G, Clark R, Zvelebil M, Griffiths GM (2001). Fas ligand is targeted to secretory lysosomes via a proline-rich domain in its cytoplasmic tail. *Journal of Cell Science*.

[28] Crncic TB, Laskarin G, Frankovic KJ (2007). Early pregnancy decidual lymphocytes beside perforin use Fas ligand (FasL) mediated cytotoxicity. *Journal of Reproductive Immunology*.

[29] Hammer A, Dohr G (2000). Expression of Fas-ligand in first trimester and term human placental villi. *Journal of Reproductive Immunology*.

[30] Aschkenazi S, Straszewski S, Verwer KM, Foellmer H, Rutherford T, Mor G (2002). Differential regulation and function of the Fas/Fas ligand system in human trophoblast cells. *Biology of Reproduction*.

[31] Jerzak M, Bischof P (2002). Apoptosis in the first trimester human placenta: the role in maintaining immune privilege at the maternal-foetal interface and in the trophoblast remodelling. *The European Journal of Obstetrics & Gynecology and Reproductive Biology*.

[32] Bai X, Williams JL, Greenwood SL, Baker PN, Aplin JD, Crocker IP (2009). A placental protective role for trophoblast-derived TNF-related apoptosis-inducing ligand (TRAIL). *Placenta*.

[33] Siano A, Tonarelli G, Imaz MS (2010). Bactericidal and hemolytic activities of synthetic peptides derived from granulysin. *Protein and Peptide Letters*.

[34] Manning WC, O’Farrell S, Goralski TJ, Krensky AM (1992). Genomic structure and alternative splicing of 519, a gene expressed late after T cell activation. *Journal of Immunology*.

[35] Hanson DA, Kaspar AA, Poulain FR, Krensky AM (1999). Biosynthesis of granulysin, a novel cytolytic molecule. *Molecular Immunology*.

[36] Chung WH, Hung SI, Yang JY (2008). Granulysin is a key mediator for disseminated keratinocyte death in Stevens-Johnson syndrome and toxic epidermal necrolysis. *Nature Medicine*.

[37] Peña SV, Hanson DA, Carr BA, Goralski TJ, Krensky AM (1997). Processing, subcellular localization, and function of 519 (granulysin), a human late T cell activation molecule with homology to small, lytic, granule proteins. *Journal of Immunology*.

[38] Gansert JL, Kießler V, Engele M (2003). Human NKT cells express granulysin and exhibit antimycobacterial activity. *Journal of Immunology*.

[39] Mincheva-Nilsson L, Nagaeva O, Sundqvist KG, Hammarström ML, Hammarström S, Baranov V (2000). *γδ* T cells of human early pregnancy decidua: evidence for cytotoxic potency. *International Immunology*.

[40] Dieli F, Troye-Blomberg M, Ivanyi J (2001). Granulysin dependent killing of intacellular and extracellular Mycobacterium tuberculosis by Vgamma9/Vdelta2 T lymphocytes. *Journal of Infectious Diseases*.

[41] Anderson DH, Sawaya MR, Cascio D (2003). Granulysin crystal structure and a structure-derived lytic mechanism. *Journal of Molecular Biology*.

[42] Krensky AM, Clayberger C (2009). Biology and clinical relevance of granulysin. *Tissue Antigens*.

[43] Wang Z, Choice E, Kaspar A (2000). Bactericidal and tumoricidal activities of synthetic peptides derived from granulysin. *Journal of Immunology*.

[44] Ernst WA, Thoma-Uszynski S, Teitelbaum R (2000). Granulysin, a T cell product, kills bacteria by altering membrane permeability. *Journal of Immunology*.

[45] Barman H, Walch M, Latinovic-Golic S (2006). Cholesterol in negatively charged lipid bilayers modulates the effect of the antimicrobial protein granulysin. *Journal of Membrane Biology*.

[46] Walch M, Eppler E, Dumrese C, Barman H, Groscurth P, Ziegler U (2005). Uptake of granulysin via lipid rafts leads to lysis of intracellular Listeria innocua. *Journal of Immunology*.

[47] Walch M, Latinovic-Golic S, Velic A (2007). Perforin enhances the granulysin-induced lysis of Listeria innocua in human dendritic cells. *BMC Immunology*.

[48] Gamen S, Hanson DA, Kaspar A, Naval J, Krensky AM, Anel A (1998). Granulysin-induced apoptosis. I. Involvement of at least two distinct pathways. *Journal of Immunology*.

[49] Okada S, Li Q, Whitin JC, Clayberger C, Krensky AM (2003). Intracellular mediators of granulysin-induced cell death. *Journal of Immunology*.

[50] Saini RV, Wilson C, Finn MW, Wang T, Krensky AM, Clayberger C (2011). Granulysin delivered by cytotoxic cells damages endoplasmic reticulum and activates caspase-7 in target cells. *Journal of Immunology*.

[51] Kaspar AA, Okada S, Kumar J (2001). A distinct pathway of cell-mediated apoptosis initiated by granulysin. *Journal of Immunology*.

[52] Li Q, Dong C, Deng A (2005). Hemolysis of erythrocytes by granulysin-derived peptides but not by granulysin. *Antimicrobial Agents and Chemotherapy*.

[53] Pardo J, Pérez-Galán P, Gamen S (2001). A role of the mitochondrial apoptosis-inducing factor in granulysin-induced apoptosis. *Journal of Immunology*.

[54] Clayberger C, Krensky AM (2003). Granulysin. *Current Opinion in Immunology*.

[55] Tewary P, Yang D, de la Rosa G (2010). Granulysin activates antigen-presenting cells through TLR4 and acts as an immune alarmin. *Blood*.

[56] Deng A, Chen S, Li Q, Lyu SC, Clayberger C, Krensky AM (2005). Granulysin, a cytolytic molecule, is also a chemoattractant and proinflammatory activator. *Journal of Immunology*.

[57] Castiello L, Stroncek DF, Finn MW (2011). 15 kDa Granulysin versus GM-CSF for monocytes differentiation: analogies and differences at the transcriptome level. *Journal of Translational Medicine*.

[58] Zitvogel L, Guido K (2010). The multifaceted granulysin. *Blood*.

[59] Hata A, Zerboni L, Sommer M (2001). Granulysin blocks replication of varicella-zoster virus and triggers apoptosis of infected cells. *Viral Immunology*.

[60] Fleming DC, King AE, Williams AEW, Critchley HO, Kelly RW (2003). Hormonal contraception can suppress natural antimicrobial gene transcription in human endometrium. *Fertility and Sterility*.

[61] Hameed A, Fox WM, Kurman RJ, Hruban RH, Podack ER (1995). Perforin expression in endometrium during the menstrual cycle. *International Journal of Gynecological Pathology*.

[62] Winn VD, Haimov-Kochman R, Paquet AC (2007). Gene expression profiling of the human maternal-fetal interface reveals dramatic changes between midgestation and term. *Endocrinology*.

[63] Lobo SC, Huang STJ, Germeyer A (2004). The immune environment in human endometrium during the window of implantation. *The American Journal of Reproductive Immunology*.

[64] Veljkovic Vujaklija D, Gulic T, Sucic S First trimester pregnancy decidual natural killer cells contain and spontaneously release high quantities of granulysin.

[65] Laskarin G, Redzovic A, Vukelic P (2010). Phenotype of NK cells and cytotoxic/apoptotic mediators expression in ectopic pregnancy. *The American Journal of Reproductive Immunology*.

[66] Latinovic-Golic S, Walch M, Sundstrom H, Dumrese C, Groscurth P, Ziegler U (2007). Expression, processing and transcriptional regulation of granulysin in short-term activated human lymphocytes. *BMC Immunology*.

[67] Huang LP, Lyu SC, Clayberger C, Krensky AM (2007). Granulysin-mediated tumor rejection in transgenic mice. *Journal of Immunology*.

[68] Ogawa K, Takamori Y, Suzuki K (2003). Granulysin in human serum as a marker of cell-mediated immunity. *The European Journal of Immunology*.

[69] Sakai M, Ogawa K, Shiozaki A (2004). Serum granulysin is a marker for Th1 type immunity in pre-eclampsia. *Clinical and Experimental Immunology*.

[70] Larsen B, Hwang J (2010). Mycoplasma, ureaplasma, and adverse pregnancy outcomes: a fresh look. *Infectious Diseases in Obstetrics and Gynecology*.

[71] Marks E, Tam MA, Lycke NY (2010). The female lower genital tract is a privileged compartment with IL-10 producing dendritic cells and poor Th1 immunity following Chlamydia trachomatis infection. *PLoS Pathogens*.

[72] Langer N, Beach D, Lindenbaum ES (1999). Novel hyperactive mitogen to endothelial cells: human decidual NKG5. *The American Journal of Reproductive Immunology*.

[73] Nakashima A, Shiozaki A, Myojo S (2008). Granulysin produced by uterine natural killer cells induces apoptosis of extravillous trophoblasts in spontaneous abortion. *The American Journal of Pathology*.

[74] Moffett A, Regan L, Braude P (2004). Natural killer cells, miscarriage, and infertility. *The British Medical Journal*.

[75] Le Bouteiller P, Tabiasco J, Parinaud J (2007). Soluble HLA-G and embryo implantation: frequently asked questions. *Gynecologic and Obstetric Investigation*.

[76] Rouas-Freiss N, Gonçalves RM, Menier C, Dausset J, Carosella ED (1997). Direct evidence to support the role of HLA-G in protecting the fetus from maternal uterine natural killer cytolysis. *Proceedings of the National Academy of Sciences of the United States of America*.

